# Because you had a bad day: General and daily relations between reactive temperament, emotion regulation, and depressive symptoms in youth

**DOI:** 10.1371/journal.pone.0224126

**Published:** 2019-10-24

**Authors:** Marie-Lotte Van Beveren, Sofie Kuppens, Benjamin Hankin, Caroline Braet

**Affiliations:** 1 Department of Developmental, Personality, and Social Psychology, Ghent University, Ghent, East-Flanders, Belgium; 2 Social Research Methodology Group, Centre for Sociological Research, KU Leuven, Leuven, Flemish-Brabant, Belgium; 3 Department of Psychology, University of Illinois at Urbana-Champaign, Champaign, Illinois, United States of America; Nathan S Kline Institute, UNITED STATES

## Abstract

Negative emotionality (NE) and positive emotionality (PE) have repeatedly shown to act as vulnerability factors for youth depression. Less research examined the mechanisms through which these reactive temperament traits may differently confer vulnerability to depression. Based on recent integrated models of depression proposing emotion regulation as a key underlying mechanism, the current study aimed to clarify the general and day-to-day relations among temperament, emotion regulation strategies, and depressive symptoms in Dutch-speaking youth (35% boys; *M_age_* = 13.27 years, *SD* = 1.98) using a cross-sectional (*n* = 495) and a 7-day daily diary design (*n* = 469). Self-reported temperament, trait rumination, trait positive refocusing, and depressive symptoms were measured at baseline. State rumination, state positive refocusing, and depressive symptoms were further assessed daily. Whereas results revealed that NE and PE interacted in predicting baseline and daily depressive symptoms, the cross-sectional analyses provide preliminary evidence for the hypothesis that NE and PE each provide unique pathways for understanding vulnerability to depression. Additional analyses in the daily diary study showed NE to be significantly related to trajectories of state rumination. Results contribute to a more nuanced understanding of the associations between temperament, emotion regulation strategies, and depressive symptoms in youth.

## Introduction

Adolescence is a turbulent developmental period in which the rates for depressive symptoms and major depressive disorder (MDD) rise dramatically [1]. Despite the fact that early onset depression is associated with numerous adverse long-term outcomes [2–4], research on moderating and mediating factors for explaining the development and maintenance of youth depression is still insufficient. This is partially because research on vulnerabilities for youth depression has been hindered by the lack of a unifying theoretical framework. However, the numerous theories and models of depression that have been proposed previously are not mutually exclusive and can be combined to provide a clearer understanding of the risk of depression. Interestingly, researchers have recently started to articulate integrated models of depression that take biological-affective (e.g., temperament) and cognitive-behavioral factors (e.g., emotion regulation) as well as developmental differences in depression into account (e.g., [5, 6]). Inspired by these models, the present study examined emotion regulation–or the set of processes by which emotions themselves are regulated [7]–as one possible mechanism through which temperament contributes to the development of youth depressive symptoms [8, 9].

### Biological-affective models of depression: The role of temperament

Starting from theory and research on individual differences in temperament of children and adolescents, several studies have shown an association between children’s temperament and their propensity for developing psychopathology [10, 11]. Temperament refers to the biologically-based individual differences in emotional and behavioral reactivity that appear early in life and are stable across time and situations [9]. Temperamental theories typically distinguish between negative emotionality (NE) and positive emotionality (PE), two reactive temperament traits that predispose individuals to experience different levels of emotions as well as to be more or less reactive to emotions [12]. NE refers to the susceptibility to negative emotions such as depressed mood and anxiety [12] whereas PE reflects one’s reactivity to positive emotions such as cheerfulness and states of positive engagement [12]. An extensive corpus of research has consistently linked depression with higher levels of NE [13–15]. In addition, early biological-affective models of depression–such as the renowned tripartite model of depression and anxiety [16]–also highlight the role of PE as a temperamental vulnerability to depression. More specifically, the tripartite model for distinguishing between depressive and anxious states asserts that high NE serves as a *general* risk factor for psychopathology, while low PE operates as a *specific* risk factor for depression [16, 17]. This theoretical premise is further substantiated by several studies showing that low levels of PE prospectively predict depressive symptoms in children, adolescents, and adults alike [18].

Recently, researchers also started to address the dynamic interaction between NE and PE for understanding depressive symptoms. This is in line with the tripartite model [16] and with theoretical accounts positing that positive emotions can buffer against the detrimental effects of negative emotions [19]. Studies addressing the NExPE interaction have typically found high NE to be more strongly predictive of depressive symptoms at low levels of PE, in comparison to high levels of PE. This has been replicated cross-sectionally in non-clinical youth [20, 21] as well as in youth psychiatric inpatients [22] and prospectively in non-referred adolescents [15].

### Cognitive-behavioral models of depression: The role of emotion regulation

Unfortunately, the tripartite model does not provide us with a framework for understanding why and how reactive temperament traits (differentially) contribute to depressive symptoms and–despite the bulk of evidence linking temperament to depression–fewer studies have examined the mechanisms through which individual differences in reactive temperament traits lead to the development of depressive symptoms in youth. Yet, clinical developmental research has undoubtedly indicated that the effects of temperament on psychological adjustment are rarely direct [20, 23–28]. Therefore, researchers have started to integrate insights from biological-affective models with those from well-examined cognitive-behavioral vulnerability models on depression–such as the widely cited response style theory [29, 30]–which posit that emotion regulation responses to stressful life-events contribute to the development and maintenance of depressive symptoms. Emotion regulation (ER) refers to the overall set of skills and cognitive and behavioral processes used to influence the nature and intensity of the emotion, the moment and/or situation one is having this emotion, the reappraisal of the emotion, and the actual display of the emotion [31]. ER should be distinguished from ‘coping’; despite some similarities, ER and coping are distinct in numerous ways. First, ER includes *both* automatic and controlled processes while coping includes only controlled volitional processes. Second, ER includes both intrinsic processes and extrinsic processes, whereas coping is only carried out by the individual that is experiencing stress (and thus only intrinsic) [7]. Third, ER includes efforts to manage emotions under a wide range of situations and in reaction to a wide range of stimuli whereas coping exclusively refers to responses to stress [7]. Following from the definition as put forward by [31] it becomes clear that ER refers to the set of processes by which emotions *themselves* are regulated, instead of how emotions regulate something else (i.e. regulation by emotion [32]). Even though ER is an umbrella term that covers both bottom-up (reactive) ER processes such as emotional awareness (i.e., the skills to identify, explain and discern one’s own as well as others’ emotional experiences [33]) and top-down (deliberate) processes or ER strategies [34, 35], we focus solely on ER strategies in the current study. ER strategies refer to the *specific* ways in which individuals actively and goal-oriented regulate their emotions [36]. ER strategies can be broadly divided into two categories dependent on their long-term effects on affect, behavior, and cognition, and their overall association with psychopathology [37, 38]. Some ER strategies are considered adaptive as they have been related to more overall emotional wellbeing long-term, while others have been labelled maladaptive because they have been associated with more overall maladjustment and depression specifically in the long-term [37, 38].

### Integrated models of depression

Interestingly, recent models integrating biological-affective and cognitive-behavioral models postulate that over time individual differences in ER will develop in accordance with one’s reactive temperament to the extent that youth manage their emotions in a way that is consistent with their temperamental-based tolerances [5, 23]. For example, a youth high in NE may get more easily aroused in a situation that elicits negative emotions–such as receiving peer-to-peer negative feedback–in comparison to a peer high in PE. Owing to his or her temperament, it might be difficult for this youth to positive refocus or to adaptively reappraise this situation (e.g., “maybe my peer is jealous of me” or “maybe my peer feels insecure about his or herself”). In fact, this youngster may rather get stuck in a vicious cycle of negative thoughts (i.e., rumination), leading to ever-worsening negative emotions.

In following *integrated* models of depression positing that adolescent ER may actually function as a mechanism through which reactive temperament traits increase vulnerability to developing depression [5, 6], the current study proposes ER strategies as a possible pathway linking reactive temperament traits to depressive symptoms.

Due to the prominence of the response style theory [29, 30]–which posits that rumination is a core vulnerability factor in depression–empirical research on integrated models of depression so far has focused predominately on clarifying the specific role of this maladaptive ER strategy in the reactive temperament–depression relationship [38, 39]. **Rumination** can be defined as repeatedly and passively thinking about one’s negative emotions, its causes, and its consequences [40] and has been shown to mediate the effect of high levels of NE on depressive symptoms [26, 41]. These findings are in line with integrated models of depression stating that high NE is in itself a vulnerability factor to depression and, in addition, that it contributes developmentally to vulnerability, such as the tendency to ruminate [5, 40]. More specific, negative affect is theorized to engender repetitive, narrowed, and pessimistic thinking (i.e., rumination)–through its narrowing effects on the attentional scope [19]–which in turn leads to ever-worsening negative emotions and even depressive symptoms [42].

However, others also suggest that PE exerts distinct and beneficial effects on ER through the broadened attentional scope that is usually caused by positive emotions [43]. Based on empirical evidence showing that–contrary to negative emotions, which predict local biases (i.e., visual processing of local–rather than global–aspects) reflecting narrowed attention–positive emotions predict global biases (i.e., global–rather than local–visual analysis) reflecting broadened attention [44], it is theorized [45] that unlike negative emotions which restrict people’s thought-action repertory (e.g., fight or flight), positive emotions broaden people’s cognitive abilities and behavioral repertoires encouraging them to be open to new experiences and discover novel lines of thought or action [19]. Over time, these cognitive and behavioral responses in response to positive emotions may increase the tendency to use adaptive ER strategies that influence the experience of both positive *and* negative emotions elicited by one’s reactive temperament. Essentially, it can be presumed that low PE predicts depressive symptoms through a lack of adaptive ER strategies. One adaptive ER strategy that fits within such a theoretical framework because it has been demonstrated to also increase positive affect [46] is positive refocusing. **Positive refocusing** refers to thinking about joyful and pleasant issues instead of pondering about the actual (stressful) event [47]. Other than ER strategies for the regulation of *positive* emotions such as savoring (i.e., the tendency to distract or redirect attention away from positive emotion in order to reduce it [48–50]), positive refocusing is considered to be an effective strategy for regulating *negative* emotions. Positive refocusing has demonstrated to be negatively associated with depressive symptoms in adolescents [47, 51, 52], while lower levels of positive refocusing have been found to predict the recurrence of depression in adults [53]. In addition, preliminary evidence in youth revealing that problems in positive refocusing play a specific role in understanding the low PE–depressive symptoms relation [28] suggests that this ER strategy may represent a key factor in furthering our understanding on why low PE heightens risk for developing youth depressive symptoms.

Given this theoretical and empirical background, it can be hypothesized that high NE and low PE each elicit a distinct pathway through which reactive temperament traits confer basic vulnerability for depression. More specific, the different aspects of reactive temperament are assumed to define different types of ER strategies with trait NE enhancing one’s general tendency to use maladaptive ER strategies such as rumination through narrowing the attentional scope and trait PE contributing to the development of adaptive ER strategies such as positive refocusing through broadening the attentional scope. Unfortunately, depression research has largely been focusing on the role of one’s general tendency to ruminate in the NE–depression relation, leaving much of the relation between PE and youth depression remains unknown.

In addition, most studies in youth have typically used a cross-sectional design [20, 54] or a two time-point prospective design [14] utilizing trait questionnaires to assess ER. These studies generally adopt a “trait approach” to ER–defining ER as one’s generally stable tendencies to regulate one’s own emotions, suggesting that there are stable differences *between* individuals in the ER strategies they tend to use [55]. However, some basic knowledge about the use of ER strategies in the context of youth’s everyday life is lacking. First, while it is assumed that cognitive and behavioral responses to emotions indeed develop towards trait-like individual characteristics over time [56], evidence in adults suggests that there are also considerable differences *within* individuals in the daily use of ER strategies (i.e., changes from moment to moment) [57]. Yet, parallel evidence in youth is currently lacking and we cannot automatically apply this knowledge to children and adolescents. More specifically, ER strategies develop and change over the course of childhood and adolescence as children shift from being dependent on their parents for ER to being able to autonomously use ER strategies to influence the intensity and duration of emotion [58, 59]. As a result of brain maturation, youth shift from relying on basic external ER strategies, such as support seeking, to relying on internal ER strategies, such as distraction and cognitive reappraisal, which require higher cognitive control capacities [59, 60]. Hence, ER strategies develop across childhood and adolescence rather than remaining stable and trait-like. Second, it has been suggested that the momentary choice for a certain ER strategy may be influenced by emotional intensity [61]. However, it remains unclear how within-person differences in ER strategies are related to individual differences in emotional *reactivity* (i.e., reactive temperament). So, the proposition that the daily use of ER strategies is also–at least partially–driven by individual differences in reactive temperament traits has not been subjected to scrutiny thus far. Yet, it cannot be ruled out that there may be differences between the general and day-to-day relations between reactive temperament and ER strategies. It may be the case that one’s general tendency to automatically engage in rumination (trait rumination) when feeling sad or depressed as a result of one’s NE reactivity is less clear-cut in everyday life. In the context of daily life, resilient factors such as temperamental reactivity to positive emotions (PE) may play an imperative role by providing these youths with alternative ER strategies aside from rumination to cope with negative emotions from time to time. Perhaps NE and PE interact in predicting daily ER strategies: while NE may have a unique influence on whether or not someone is likely to develop an inclination to engage in rumination from a trait approach [40], it is plausible that NE and PE jointly exert their influence on the use of ER strategies in everyday life so that youth show fluctuations in the use of ER strategies from day to day. More specific, high PE may exert a buffering effect on the NE–ER relation in such a way that it reduces the extent to which rumination is used on a daily basis through broadening the scopes of attention and cognition and by initiating positive thought-feeling-action chains or ‘upward spirals’ (i.e., the reciprocal relation between positive emotions and personal recourses [62]) toward enhanced emotional wellbeing as a result of increases in daily positive affect [44]. Unfortunately, few researchers have adopted a “state approach” to ER, considering ER as a dynamic process. As a result, no study so far examined the role of the NExPE interaction for understanding state ER strategies in youth. Ideally, combining a cross-sectional design with a daily diary design could enable a more thorough examination of both the general and day-to-day relations among reactive temperament traits, ER strategies, and depressive symptoms.

### The current study

The central goal of the current study is to examine both the general and daily relations among reactive temperament traits, ER strategies, and depressive symptoms in youth using two different designs: a cross-sectional and a 7-day daily diary design. Based on integrated models of depression [5, 6], the current study has three aims. First, to replicate the findings pertaining to the well-established relation between NExPE and depressive symptoms in youth [15, 20–22] by examining whether NE and PE interact in predicting both cross-sectional–as well as daily depressive symptoms in youth. Second, to examine the mediating role of trait ER strategies in the reactive temperament–depressive symptoms relation in the cross-sectional study. Third, to explore the effects of NE and PE, and particularly their interaction, in understanding the state rumination and state positive refocusing in the daily diary study. Based on prior work [15, 20–22, 63, 64], we hypothesize that NE and PE will interact in explaining baseline depressive symptoms in the cross-sectional study, as well as in explaining daily symptoms in the daily diary study. Second, starting from a trait approach to ER [26, 28, 41], we hypothesize that distinct mechanisms will arise in the reactive temperament–depressive symptom relation with NE and PE each predicting baseline depressive symptoms through their unique effects on specific trait ER strategies in the cross-sectional study. More specifically, we hypothesize that trait rumination will significantly contribute in explaining the specific relation between NE–but not PE–and baseline depressive symptoms and that trait positive refocusing will significantly contribute in explaining the PE–but not the NE–symptoms relation. Lastly, given that the current study is the first to explore the effects of NE and PE on daily ER strategies, we are not able to substantiate our third hypothesis with empirical evidence. Nonetheless, starting from a state approach we hypothesize that NE and PE will interact in predicting state rumination and state positive refocusing. Given the gender and age differences in youth depressive symptomatology and ER strategies [5, 65], gender and age were included as covariates of no interest in all analyses to filter out the possible confounding effects these variables.

## Method

### Participants

Participants were 531 Dutch speaking youth (35% boys) within an age range from 9 to 17 years (*M_age_* = 13.27, *SD* = 1.98). All participants lived in the Flemish region of Belgium. The majority of the families in the current sample were of upper middle (38%) or middle class (45%) socioeconomic status (SES) based on the parents' educational level and current occupation [66]. Furthermore, 12.5% reported moderate depressive symptoms at baseline, whereas an additional 7.3% of youth reported severe depressive symptoms [67]. All participants came from different families.

### Procedure

The current study is part of larger research project on emotional wellbeing in youth at the Clinical Developmental Psychopathology Department of Ghent University. The protocol of the study was approved by the Ethical Committee of the Faculty of Psychology and Educational Sciences at Ghent University. Third-year psychology students were instructed to recruit two Dutch-speaking participants between age 10 and 16 of the Belgian–Flemish population with which they were not emotionally involved (no family, friends, etc.) and visit them at home. No other in- or exclusion criteria were put forward. After obtaining both child and parental written informed consent, participants were asked to fill out a paper and pencil test battery comprising measures of overall psychopathology, reactive temperament, and trait ER strategies in a separate room at home. During the assessment, one of the students remained present in the room to answer questions pertaining to the test battery. After completing the home-assessment, participants were asked to participate in the 7-day daily diary study (e.g., [68, 69, 70]). In the daily diary study, participants were instructed to fill out a shortened test battery on a secure online platform hosted by the Department of Developmental, Personality, and Social Psychology on their own computer at home, starting from the following Monday. The online test battery consisted of three scales assessing (daily) depressive symptoms, state rumination, and state positive refocusing. Participants were told to complete the diary on a daily basis before bedtime. The participants’ parents were given standardized instructions and received a daily reminder by e-mail or by phone at the end of the day during that week. Participation was not remunerated.

Of all of the participants, 36 youth did not fill out the self-report questionnaires during the home visit. These youths did not significantly differ from the current sample in terms of gender and age (all *p*s *≥* .789). Furthermore, 62 children did not fill out the daily diary because they found it too time-consuming. These youths did not significantly differ from the remaining sample on gender, age, NE, PE, and depressive symptoms at baseline (all *p*s *≥* .142).

Preliminary analyses of the cross-sectional data suggested that the percentage of missing data ranged between 0% and 1.4% per item. Comparison of means and covariances of all variables using Little [71] MCAR-test produced a normed χ^2^ (χ^2^/df) of 1.05, *p* > .30, indicating that the data were likely missing completely at random [72]. Consequently, missing item values were imputed following the expectation maximization (EM) algorithm available in SPSS 23 [73].

It is expected that in daily diary studies there will be missing data over the 7 days, as was the case in the current study. Participation across weekly assessment was acceptable. The average number of weekly assessments completed was 5.81 (*SD* = 1.85) out of a possible 7. In total, 59.4% (279) of participants completed all seven assessments, 14% (66) completed six assessments, 6.8% (32) completed five assessments, 4% (19) completed four assessments, 5.7% (27) completed three assessments, 4.3% (20) completed two assessments and 5.7% (27) completed one assessment. In order to retain all available information, a specific time point was dropped from the analyses when one time-point was missing instead of dropping a participant from the entire analyses. Comparison of youth that completed all daily assessments of the diary study and youth who didn’t comply revealed that there were no significant differences between youth regarding gender, age, NE, PE, and depressive symptoms in the cross-sectional study, as well as daily levels of depressive symptoms in the daily diary study (all *p*s *≥* .253).

### Cross-sectional measures

#### Reactive temperament

The trait version of the Positive and Negative Affectivity Schedule for Children (PANAS-C) [74] was used to assess reactive temperament. The PANAS-C has repeatedly been used as a measure of reactive temperament in youth [14, 21, 28, 54, 64, 75, 76] and asks participants about the *general* experience of emotion rather than a *specific*–more recent–timescale. The PANAS-C is a self-report instrument for children aged 7 to 14 years that contains 30 emotion-items. Participants rate the extent to which they *usually* experience each specific emotion on a 5-point Likert scale. The questionnaire consists of two subscales that assess negative emotionality (NE; 15 items) and positive emotionality (PE; 12 items). The PANAS-C has demonstrated good convergent and discriminant validity with scores on child and adolescent self-reports of depression and anxiety [74, 77]. Cronbach’s alpha’s in the current study were α = .86 and α = .78 for the NE and PE subscale respectively.

#### Emotion regulation strategies

Trait rumination and trait positive refocusing were assessed using two subscales of the FEEL-KJ [78, 79]. The FEEL-KJ is a 90-item self-report measure assessing various ER strategies in response to anger, fear, and sadness in youth aged 8 to 18. Participants rate each item on a 5-point Likert scale from (1) “almost never” to (5) “almost always”. In the current study, participants filled in the items for the ER scale “*rumination”* and the ER scale *“positive refocusing”*. Both scales consist out of 6 items and total scores comprise the general use of these ER strategies in response to anger, fear, and sadness. The FEEL-KJ has proven to be a valid and reliable questionnaire in children and adolescents [79, 80]. Internal consistency for the separate subscales in the current study was acceptable [81] with Cronbach’s α = .68 for trait rumination and α = .82 for trait positive refocusing.

#### Depressive symptoms

The Child Depression Inventory (CDI) [82], Dutch version by [83] was used to measure baseline depressive symptoms. The CDI is a 27-item self-report measure for youth aged 7 to 17 years to assess cognitive, affective, and behavioral symptoms of depression. Each item comprises three response options, which vary in severity, and are rated on a 3-point scale; children and adolescents select the one that characterized them best during the past 2 weeks. The CDI shows good psychometric qualities in terms of internal consistency and test-retest reliability in non-clinical youth [83]. Cronbach’s alpha in the current sample was α = .85.

### Daily measures

#### State emotion regulation

State rumination and state positive refocusing were assessed using an adapted version of the “*rumination”* and the *“positive refocusing”* FEEL-KJ subscales [78, 79]. Each strategy was measured using six items that began with “When I felt angry/scared/sad today” and ended with “I wondered why I was feeling angry/scared/sad” and “I couldn’t get it out of my head./I couldn’t stop thinking about it” (state rumination) and “I thought about the things that make me happy.” And “I thought about happy things” (state positive refocusing). Each item was rated on a 5-point Likert scale from (1) “almost never” to (5) “almost always” to indicate how they regulated their anger, fear, and sadness that day. For the seven subsequent within day Cronbach’s alpha ranged between .70 and .82 for state rumination and .88 and .96 for state positive refocusing.

#### Daily depressive symptoms

The Centre for Epidemiologic Studies Depression Scale Short Form (CES-D SF) [84] was used to assess daily depressive symptoms during one week. The CES-D comprises nine items of the original 20-item measure developed by [85], which has been adapted for youth ages 6–17 years [86]. Similar to the prospective diary study of [27], the items were modified in the current study to assess depressive symptoms on a daily basis by asking participants to rate how they felt and behaved during that particular day. Responses ranged from (0) “rarely or none of the time” to (3) “most or all of the time” for items such as, “I had trouble keeping my mind on what I was doing” and “I felt sad”. The CES-D has shown strong convergent validity with other depression measures [85] and good psychometric properties for adolescents [86]. In the current study, within day Cronbach’s alpha ranged between .82 and .85 for the seven subsequent days.

### Data-analytic strategy

#### Cross-sectional analyses

First, regression analyses were conducted through multiple linear regression analysis (MLR) to test our first hypothesis i.e., whether NE and PE interact in predicting baseline depressive symptoms. Second, we ran two models to test our second hypothesis, i.e., whether trait rumination and trait positive refocusing differently contribute in explaining the temperament–baseline depressive symptoms relation. Following [87], the proposed models were tested using bootstrapping as provided by the PROCESS macro, an add-on utility for SPSS for conditional process modelling [88], which is a nonparametric resampling procedure that employs a bias-corrected bootstrap method with 1000 resamples to derive 95% confidence intervals (CI) for the indirect effects. Baseline depressive symptoms were entered as the dependent variable (DV), reactive temperament traits were successively entered as the independent variable (IV), trait rumination and trait positive refocusing were separately entered as the mediator (M) to test the two distinct pathways for understanding baseline depressive symptoms. All variables were standardized prior to computing our analyses.

#### Daily diary analyses

Data were analysed within a multilevel framework using MLwiN [89] because the seven consecutive days (Level 1) were nested within the individuals (Level 2). In following [90] recommendations, variables at Level 1 reflecting within-youth predictors were centered around the individual’s mean (group-mean centered) [90, 91] in order to eliminate the influence of Level 2 differences in the predictors for the analyses. Predictors at Level 2 reflecting between-youth predictors were centered around the grand mean (grand-mean centered) to improve the interpretation of the intercept values [90].

Prior to our main analyses, random intercept only models were used to estimate the variance partitioning coefficient (VPC), which reflects the proportion of variance in depressive symptoms, state rumination, and state positive refocusing situated between youth (Level 2). Second, random slope models were built by adding Time as a Level 1 predictor (testing whether on average there was change over time) and modelling the coefficient of Time random at Level 2 (testing whether the growth rate varied between individuals). To test our first hypothesis, i.e., whether NE and PE interact in predicting daily levels (i.e., intercepts) and trajectories (i.e., slopes) of depressive symptoms, baseline reactive temperament traits, their interaction, as well as cross-level interactions (temperament x time), were added to the random slope model for depressive symptoms. Next, in the interests of our third aim, i.e. to explore whether NE and PE interact in predicting the trajectories in state ER strategies, baseline reactive temperament traits, their interaction, as well as cross-level interactions, were included in the random slope models for state rumination and state positive refocusing consecutively to explore the role of NE and PE, as well as their interaction, in explaining the daily levels (i.e., intercepts) as well as (i.e., slopes) trajectories of state rumination and state positive refocusing respectively.

## Results

Means, standard deviations, and correlations between all variables are shown in Table 1. Mean scores of all variables in the current sample are comparable to findings of previous studies in youth samples [79, 82, 83, 92, 93]. All variables were correlated in the hypothesized direction.

**Table 1:** Variable correlations and descriptives.

Variables	1	2	3	4	5	6	7	M (SD)	Min	Max
1. NE								33.29 (8.37)	16.00	61.00
2. PE	-.19**							44.96 (6.00)	15.00	60.00
3. CDI	.57**	-.48**						8.60 (5.85)	0.00	42.00
4. Positive refocusing	-.12**	.30**	-.28**					21.49 (4.59)	6.00	30.00
5. Rumination	.39**	-.08	.31**	.06				18.28 (4.36)	6.00	30.00
6. CES-D	.41**	-29**	.59**	-25**	.28**			4.75 (3.53)	0.00	20.43
7. State positive refocusing	-.15*	.21**	-.23**	.51**	.05	-.17**		18.30 (6.67)	6.00	30.00
8. Sate rumination	.17**	-.04	.20**	.03	.44**	.41**	.36**	14.91 (4.58)	6.00	29.43

*Note*: NE = *negative emotionality*; PE = *positive emotionality*; CDI = *baseline depressive symptoms*; CES-D = *mean of daily depressive symptoms over the 7 consecutive days*

* *p* < .05

** *p* < .01

### Cross-sectional analyses

#### Do NE and PE interact in predicting baseline depressive symptoms?

**Fig 1:**
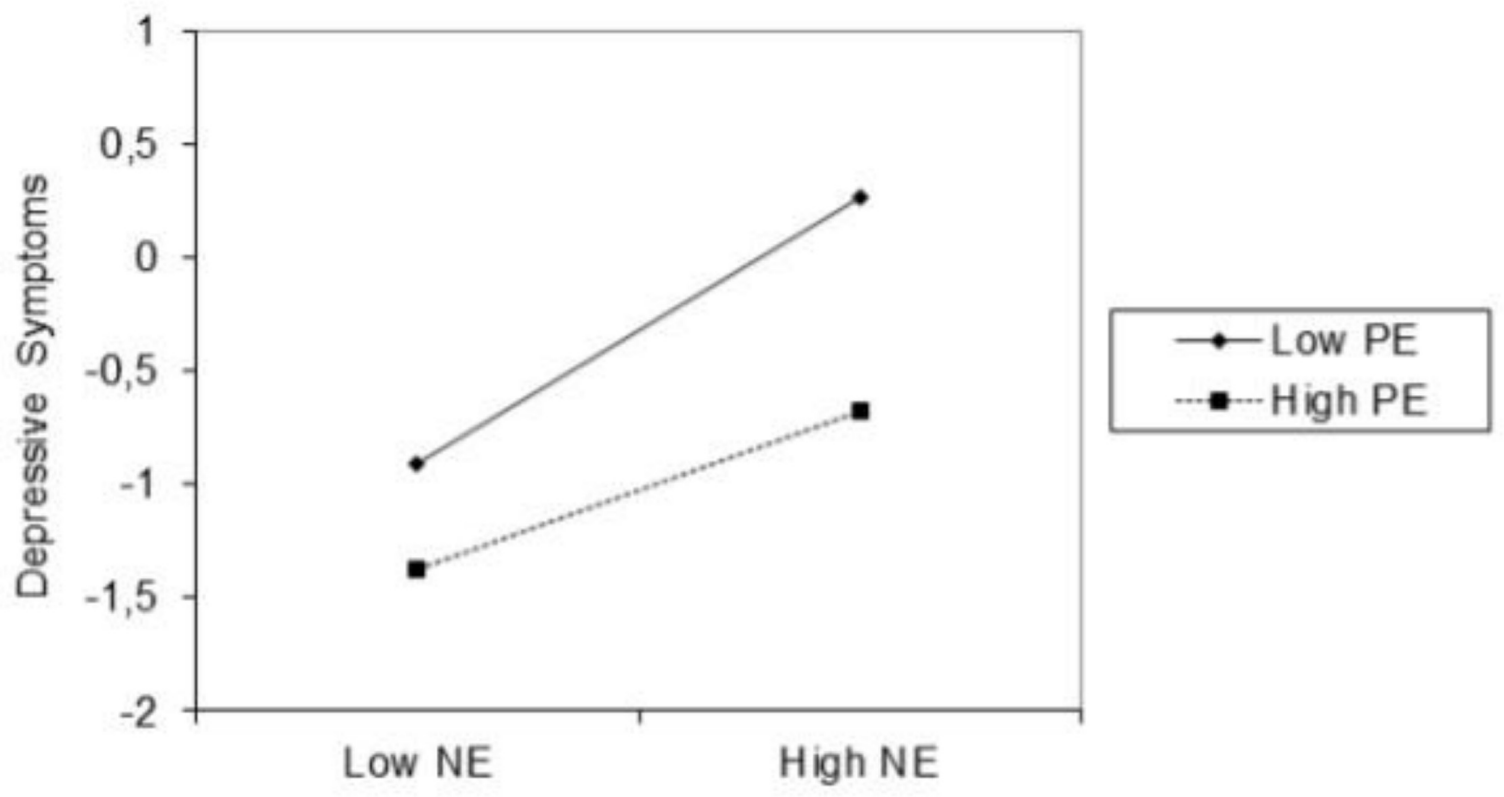
The interaction effect between negative emotionality (NE) and positive emotionality (PE) in the prediction of baseline depressive symptoms .

Results (Table 2; Model 1) support our first hypothesis that NE and PE interact in predicting baseline depressive symptoms (*β* = -.16, *t* (489) = -4.75, *p* < .001, sr^2^ = .02). As visualized in Fig 1, the significant interaction was interpreted by plotting the simple regression lines for the high (+1SD) and low (-1SD) values of PE. Although NE was significantly and positively associated with baseline depressive symptoms at both levels of PE, the effect was less pronounced at high PE (*β* = .35, *t* (489) = 7.80, *p* < .001) compared to low PE (*β* = .58, *t* (489) = 15.62, *p* < .001).

**Table 2:** Cross-sectional analyses for the mediation models including trait rumination (Model 2 and 3) and trait positive refocusing (Model 4 and 5).

	Model 1 B (SE(B))	Model 2 B (SE(B))	Model 3 B (SE(B))	Model 4 B (SE(B))	Model 5 B (SE(B))
	DV = CDI	DV = Trait rumination	DV = CDI	DV = Trait positive refocusing	DV = CDI
Gender	-.05 (.02)**	-.07 (.02)**	-.05 (.02)**	.03 (.02)	-.05 (.02)**
Age	.06 (.02)**	.07 (.02)**	.05 (.02)**	-.04 (.02)	.05 (.02)**
NE	.47 (.03)***	.40 (.04)***	.46 (.04)***	-.08 (.05)	.49 (.03)***
PE	-.35 (.03)***	.03 (.04)	-.37 (.03)***	.27 (.05)***	-.34 (.03)***
NExPE	-.12 (.03)***	-.02 (.03)		-.03 (.03)	
Trait rumination			.08 (.04)*		
Trait positive refocusing					-.11 (.03)**
**R^2^**	.51	.18	.49	.10	.50
**Change statistics**	*F* Change (3, 489) = 156.94***	*F* Change (3, 489) = 31.91***	*F* Change (1, 489) = 5.62*	*F* Change (3, 489) = 15.04***	*F* Change (1, 489) = 10.38 **

*Note*: NE = *negative emotionality*; PE = *positive emotionality*; CDI = *baseline depressive symptoms*

* *p* < .05

** *p* < .01

*** *p* < .001

#### Does trait rumination contribute in explaining the specific relation between NE and baseline depressive symptoms?

**Fig 2:**
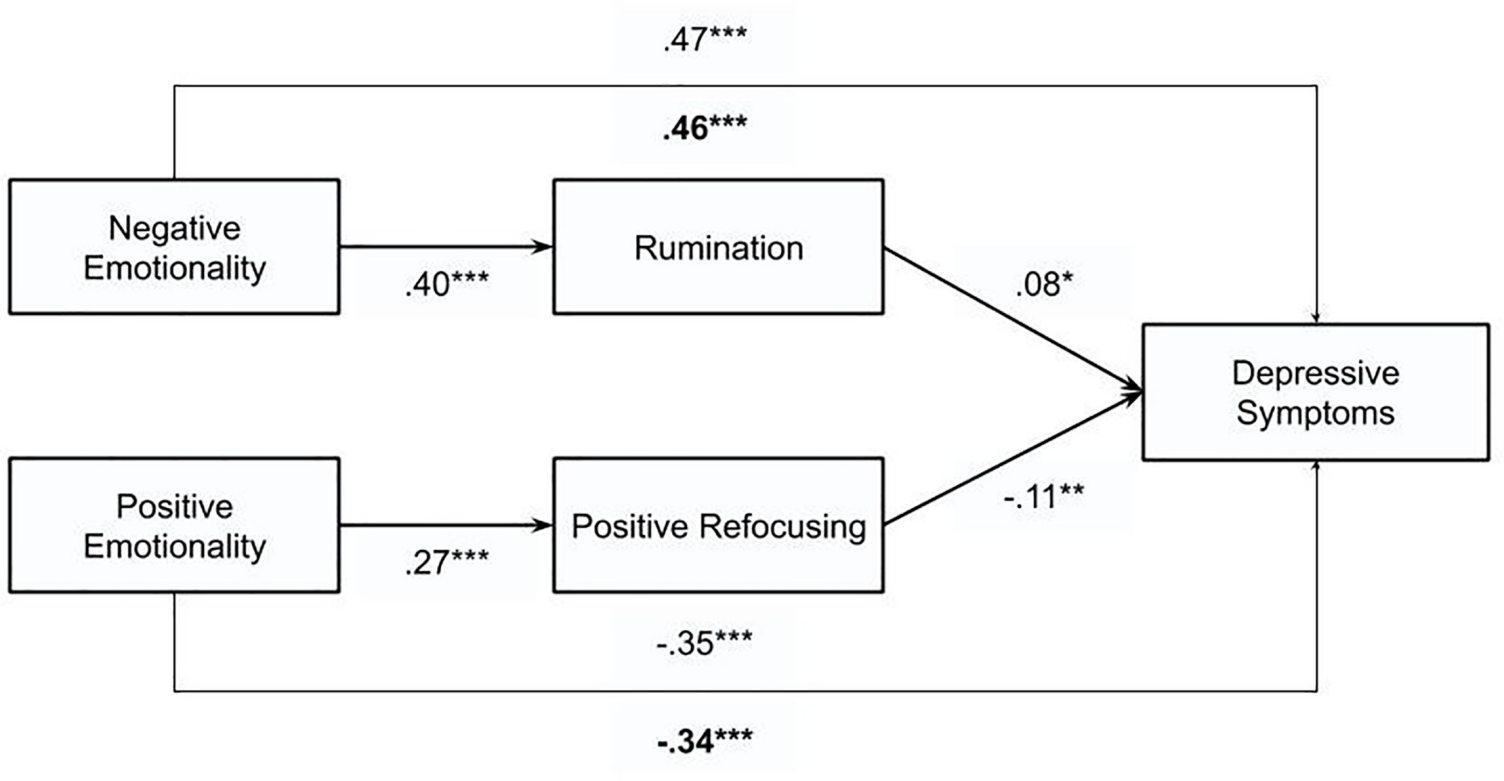
Results for the cross-sectional mediation analyses.

Consistent with our second hypothesis, results (Fig 2; Table 2; Model 2 and 3) revealed that the indirect effect of NE on depressive symptoms through trait rumination was significant (*β* = .03, *SE* = .01, *z* = 2.29, 95% CI [.01,.06], *p* = .022), suggesting that high levels of NE predicted higher baseline depressive symptoms through its effect on trait rumination, i.e., a larger tendency to use this strategy. Notably, the direct effect of NE on baseline depressive symptoms remained significant, indicating a partial mediation. No significant relation was found between PE and trait rumination (*p* = .485).

#### Does trait positive refocusing contribute in explaining the specific relation between PE and baseline depressive symptoms?

Also consistent with our second hypothesis, results (Fig 2; Table 2; Model 4 and 5) revealed that the indirect effect of PE on depressive symptoms through trait positive refocusing was significant (*β* = -.03, *SE* = .01, *z* = -2.80, 95% CI [-.06, -.01], *p* = .005), leading us to conclude that higher PE predicted lower baseline depressive symptoms through its effect on trait positive refocusing, i.e., a lower use of this strategy. Markedly, the direct effect of PE on depressive remained significant, indicating a partial mediation. No significant relation was found between NE and trait positive refocusing (*p* = .097).

In short, results pertaining our second aim support the hypothesis that NE and PE each provide unique pathways in explaining vulnerability to depression: trait rumination partially explained the specific relation between NE and baseline depressive symptoms, whereas trait positive refocusing partially explained the PE–but not the NE–symptoms relation.

### Daily diary analyses

The VPC derived from the random intercept only models indicated that 61.6% of the variance in daily depressive symptoms was situated between youth. With regard to state rumination and state positive refocusing, the VPC revealed that 66.4% and 75.0% of the variance reflected between-youth differences respectively. In addition, random slope models were fitted for core study variables before running the main analyses. Results from these models (Table 3) revealed that daily depressive symptoms (Model 1) as well as state rumination (Model 1R) and state positive refocusing (Model 1PR) generally decreased as the week progressed, as indicated by the negative time-coefficient.

**Table 3:** Daily depressive symptoms, state rumination (R) and state positive refocusing (PR) as a function of reactive temperament.

	Model 1	Model 2	Model R1	Model R2	Model PR1	Model PR2
**Fixed parameters**						
Constant	5.58 (.20)***	5.48 (.25)***	16.32 (.21)***	11.72 (1.40)***	18.69 (.24)***	15.99 (1.84)***
Time	-.18 (.03)***	-.18 (.03)***	-.45 (.04)***	-.46 (.04)***	-.18 (.05)***	-.20 (.05)***
**Initial status**						
Gender		.02 (.30)		1.00 (.42)*		1.38 (.55)*
Age		.31 (.08)***		.30 (.10)**		0.134 (.14)
NE		.18 (.02)***		.10 (.02)***		-0.11 (.03)***
PE		-.19 (.03)***		.02 (.04)		.23 (.05)***
NE x PE		-.01 (.00)*		-.00 (.00)		-.00 (.00)
**Rate of change**						
NE x time		-.01 (.00)		-.01 (.01)**		.00 (0.01)
PE x time		.01 (.01)*		-.00 (.01)		.01 (.01)
NE x PE x time		-.00 (.00)		-.00 (.00)		-.00 (.00)
**Random Parameters**						
$\sigma_{u0}^{2}$	13.61 (1.15)***	8.38 (.80)***	16.33 (1.34)***	14.55 (1.22)***	28.18 (2.22)***	25.38 (2.03)***
*σ*_*u*0*u*1_	-.53 (31)***	-.33 (.13)**	-.13 (.18)	.01 (.17)	.62 (.30)*	.57 (.29)*
$\sigma_{u1}^{2}$	.17 (.03)***	.15 (.03)***	.34 (.04)***	.33 (.05)***	.72 (.08)***	.71 (.08)***
$\sigma_{e0}^{2}$	6.54 (.22)***	6.53 (.22)***	6.77 (.23)***	6.77 (.23)***	8.87 (.30)***	8.87 (.30)***
**Deviance**	13616.04	13429.67	13959.28	13916.75	14911.67	14866.97

*Note*: The reference category for gender was male. NE = *negative emotionality*; PE = positive emotionality; $\sigma_{u0}^{2}$ = between-person variance; $\sigma_{u1}^{2}$ = variance in the slope; $\sigma_{e0}^{2}$ = within-person variance; *σ*_*u*0*u*1_ = *covariance between random intercept and random slope;* Model 1 = *random slope model for depressive symptoms;* Model 2 = *inclusion of gender*, *age*, *reactive temperament traits and the NExPE interaction term to Model 1*; Model R1 = *random slope model for state rumination;* Model R2 = *inclusion of the main effects of reactive temperament traits and the NExPE interaction term to Model R1;* Model PR1 = *random slope model for state positive refocusing;* Model PR2 = *inclusion of the main effects of reactive temperament traits and the NExPE interaction term to Model PR1*.

* *p* < .05

** *p* < .01

*** *p* < .00

#### Do NE and PE interact in explaining daily depressive symptoms?

**Fig 3:**
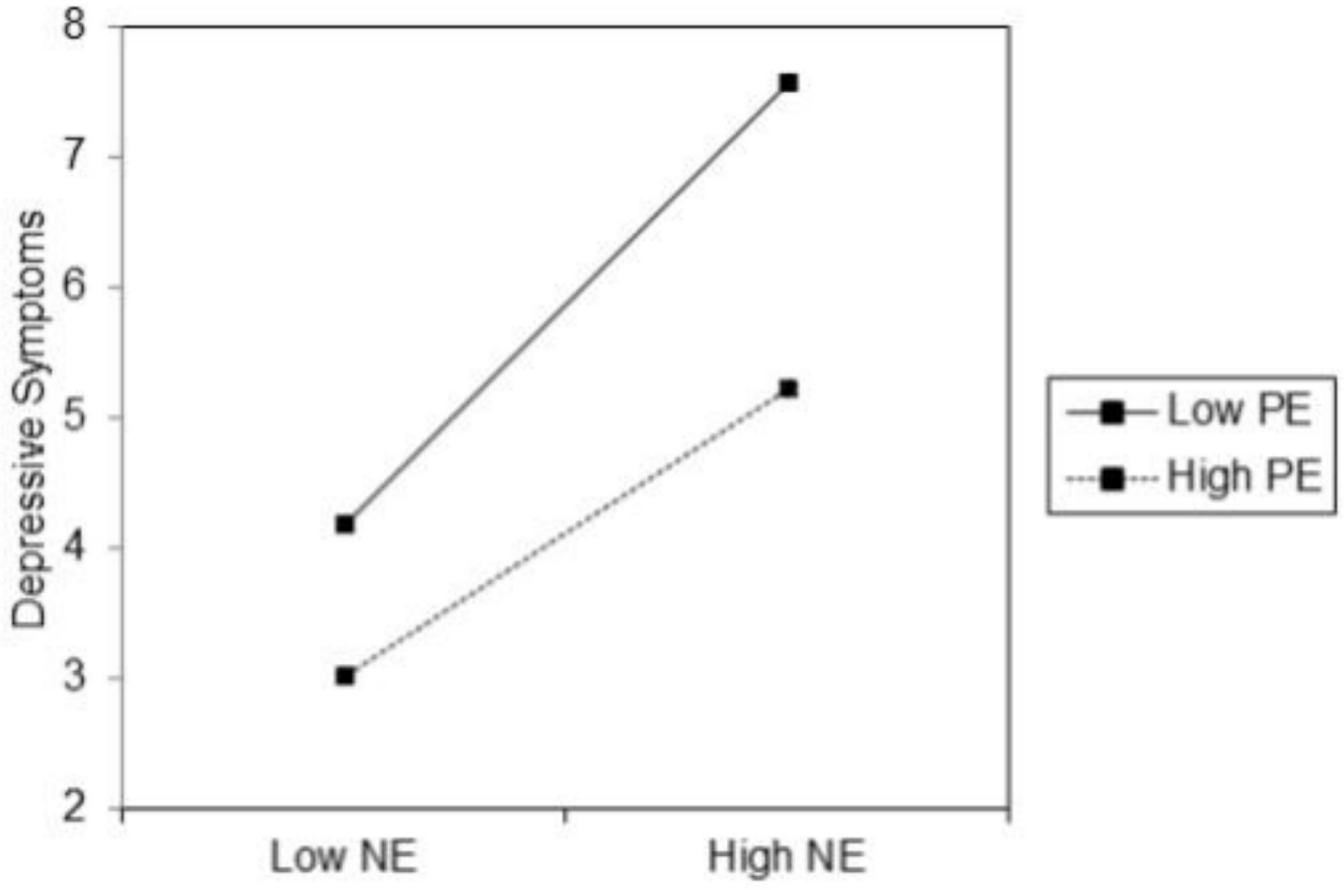
The interaction effect between negative emotionality (NE) and positive emotionality (PE) in the prediction of daily depressive symptoms .

Consistent with our first hypothesis, results (Table 3; Model 2) revealed that NE and PE interact in explaining daily (i.e., intercepts) depressive symptoms (*χ*^2^(1) = 4.29, *p* = .038). Fig 3 shows the prediction plot for depressive symptoms at day 1 for youth low (-1SD) or high (+1SD) in NE in combination with low (-1SD) or high (+1SD) PE levels. This plot shows that the effect of high levels of NE was less pronounced at high PE compared to low PE. Combined, NE and PE, as well as their interaction, explained 34.17% of the variance in daily depressive symptom. Contrary to our expectations, no significant effects were found for the NExPE interaction on trajectories (i.e., slopes) of depressive symptoms (*p* = .950). Surprisingly, further inspection of the main effects of NE and PE revealed while NE did also not significantly relate to trajectories of depressive symptoms (*p* = .145), PE did significantly predict these trajectories (*χ*^2^(1) = 6.10, *p* = .014). More specifically, youth high in PE showed a steeper decrease in symptoms throughout the week. Temperament explained 11.77% of the variance in the trajectories of depressive symptoms.

#### Do NE and PE interact in explaining state rumination?

Results (Table 3; Model R2) pertaining our third aim revealed that NE, but not PE, was significantly and positively associated with the intercepts of state rumination (*χ*^2^(1) = 16.87, *p* < .001) showing that youth high in NE reported a higher use of state rumination at day 1. Temperament explained 10.90% of the variance in daily levels of state rumination. Additionally, NE exerted a significant effect on trajectories (i.e., slopes) of state rumination (*χ*^2^(1) = 6.65, *p* = .01), revealing that youth high in NE showed a slower decrease in the use of rumination throughout the week. Nor the NExPE interaction, nor PE were significantly predictive for trajectories of state rumination (all *p*s ≥ .260). Temperament explained 2.94% of the variance in trajectories of state rumination.

#### Do NE and PE interact in explaining state positive refocusing?

Results (Table 3; Model PR2) revealed that PE was significantly and positively associated with the intercepts of state positive refocusing (*χ*^2^(1) = 22.95, *p* < .001), showing that youth high in PE reported a higher use of state positive refocusing on day 1. Temperament explained 9.94% of the daily levels of state positive refocusing. To our surprise, NE was also significantly related to the intercepts of state positive refocusing (*χ*^2^(1) = -10.33, *p* = .001) so that youth high in NE reported a lower use of positive refocusing on the first day. Nor the NExPE interaction, nor NE and PE were significantly predictive for trajectories (i.e., slopes) of state positive refocusing (all *p*s ≥ .089). Temperament explained 8.06% of the variance in trajectories of state positive refocusing.

## Discussion

The current study aimed to further clarify the general and daily relations among reactive temperament traits, ER strategies, and depressive symptoms in youth age 9 to 17 years using two different designs: a cross-sectional design and a 7-day daily diary design. Overall, the current study had three main findings pertaining to our aims. First, as predicted, NE and PE interacted in explaining baseline depressive symptoms in the cross-sectional study, as well as in explaining daily symptom levels in the daily diary study. Second, results confirm our hypothesis that NE and PE each predict baseline depressive symptoms through their unique effects on trait ER strategies in the cross-sectional study. Third, whereas NE and PE did not interact in explaining within-person changes in state ER strategies in the daily diary study, the exploratory analyses revealed that NE was significantly related to trajectories of state rumination. These results contribute to a more nuanced understanding of the relation between reactive temperament traits and depressive symptoms in youth as previously posited by biological-affective and cognitive-behavioral models of depression, suggesting that ER strategies may function as a mechanism through which reactive temperament traits increase vulnerability to depression.

The first aim sought to replicate previous findings pertaining to the relation between reactive temperament traits and depressive symptoms in adolescents. Consistent with our hypotheses, NE and PE interacted in predicting higher baseline and daily depressive symptoms suggesting that high PE serves as a buffer in the positive relation between high NE and depressive symptoms [15, 20–22]. By reducing the impact of negative affect, high PE may thus constitute a resilience factor that buffers against depressive symptoms [94]. However, it should be of note that the additional amount of variance that was explained by adding the NExPE interaction to the model for explaining depressive symptoms was rather low in both studies. Furthermore, the exploratory analyses revealed that, contrary to what is to be expected from a state approach, the NExPE interaction did not appear to be of significant importance for predicting trajectories of depressive symptoms in the daily diary study. In fact, our findings suggest that temperamental PE, rather than the dynamic interplay between NE and PE, is essential to a more profound understanding of these trajectories.

More specifically, the analyses revealed that PE, but not NE, predicted prospective trajectories of depressive symptoms across a one-week period, revealing that with higher levels of PE, depressive symptoms showed a steeper decrease throughout the week. This tentative finding advocates for a more nuanced view on the role of NE for understanding depressive symptoms and further calls for differentiating the effects of positive emotions from those of negative emotions [62]. More specifically, it can be suggested that being high in PE may make youth more reactive to and aware of the positive things in everyday life. The subsequent positive emotions may not only feel good in the present, but also increase the likelihood that one will feel good in the future [44]. Hence, the positive emotions resulting from facing more positive experiences throughout the day may provide positive thought-feeling-action chains (i.e., ‘upward spirals’) for youth high in PE [44], which in turn contribute substantially to one’s overall mood and emotional wellbeing [62, 95]. Despite the fact that evidence in adults collected through the experience sampling method (ESM) [96] advocates for the emotion context insensitivity view of MDD which characterizes its core affective pathology in terms of a generalized blunting of both negative and positive affect reactivity to negative and positive stimuli [97], the current study only provides partial evidence for such a view in non-clinical youth. In fact, our findings are in line with the large body of research documenting reward deficits in depression (i.e., reduced sensitivity to rewarding outcomes) [98, 99] and fit within the positive attenuation view for understanding MDD [100] which characterizes its core affective pathology in terms of reduced positive affect reactivity to positive stimuli and thus predicts that depressed individuals with the lowest reactivity to positive stimuli will have the worst MDD course.

The second aim sought to clarify the relation between reactive temperament traits, trait ER, and depressive symptoms in youth. Based on integrated models of depression [5, 6] it was hypothesized that the overall association between reactive temperament traits and youth’s depressive symptoms can be explained by trait ER strategies. Results of the cross-sectional study provide preliminary evidence for our hypothesis that NE and PE each provide unique pathways in defining basic vulnerability to depression. More specifically, trait rumination explained the specific relation between NE and baseline depressive symptoms, whereas trait positive refocusing explained the PE–but not the NE–symptoms relation. This is consistent with previous studies assessing ER strategies by using trait questionnaires, which repeatedly found evidence for the mediating role of trait rumination in the relation between NE and youth depressive symptoms both cross-sectionally [14, 54] and longitudinally [14, 25, 41]. Our findings add to the scarce literature on PE and adaptive ER strategies, indicating that PE may similarly predict depressive symptoms through a lack of adaptive ER strategies such as trait positive refocusing [28] and are commensurate with the findings of a recent study of [94] conducted in adults, suggesting that there may be different pathways that underlie depressive symptoms. [94] suggest that for some individuals, one pathway to depression may be heightened reactivity to stress or negative affect, whereas for others, this may be initiated by a diminished favourable impact of positive affect. Our findings further built on this proposition by suggesting that the temperament-related differential use of trait ER strategies may be one mechanism underlying these distinct pathways.

The third and last research line aimed at clarifying the relation between temperament and the daily use of ER strategies in youth given that fewer studies have adopted a “state approach” to ER strategies. Whereas the exploratory analyses did not provide evidence for the assertion that NE and PE interact in predicting state ER strategies, several interesting findings emerged. First, results revealed that NE significantly contributed to explaining the trajectories of state rumination, showing that with higher levels of NE the use of state rumination showed a less steep decrease as the week progressed. This finding lines up with numerous previous studies adding further support to the robust predictive role of NE for the use of rumination in youth [13, 14, 41, 54] and suggest that PE does not play a crucial role in understanding the general, nor the daily use, of this ER strategy. Furthermore, this result adds to the previous findings of studies using the EMA in clinical youth (9–13 years [101]) revealing that–in comparison to healthy controls–youth diagnosed with an anxiety disorder are not only more reactive to stressful events (which may be elicited by their heightened levels of NE) but are also less effective at down-regulate negative emotions as indicated by a higher use of state rumination.

Second, whereas PE, but not NE, appeared to be significantly related to *trait* positive refocusing in the expected direction, both NE and PE were significantly associated with *state* positive refocusing. These findings suggest that, whereas one’s general tendency to incorporate positive refocusing into one’s ‘trait ER strategy repertoire’ may be mainly determined by PE, the daily use of this strategy may be influenced by both temperament traits. Hence, the specific effects of NE and PE on the daily use of ER strategies may not be as clear-cut. Despite the effects of one’s temperament on trait and intercepts of state positive refocusing, the exploratory analyses suggest that neither the NExPE interaction, nor NE and PE significantly contributed to explaining the trajectories of state positive refocusing in the daily diary study. A possible explanation for this tentative finding may be found in the fact that we used a non-clinical sample. Indeed, both mean baseline CDI scores and daily CES-D scores revealed that levels of depressive symptoms were rather low among our sample. It may be that adaptive ER strategy use is more stable among non-clinical youth, whereas clinically depressed youth show more fluctuations in the use of adaptive ER skills. For example, it may be that clinically depressed youth use little to no adaptive ER strategies when they have a “bad day”, whereas they do (try to) adopt such strategies when they are feeling better and have more energy.

Although not of primary interest, results revealed that age was significant in predicting depressive symptoms in both studies–a finding that aligns with the marked increase in prevalence rates of depression during adolescence [102]. Consistent with previous work [103], age was also significantly associated with trait and state rumination indicating that the use of this ER strategy increases with age. This increase in rumination may be the result of the attainment of a more fully conscious, self-directed, and self-regulating mind during adolescents [104] and the heightened stress levels that accompany the adolescent period [30]. Our finding that gender significantly predicted baseline depressive symptoms reflects the well documented gender difference in adolescent depression [5, 105, 106], showing that girls experience increases in depressive symptoms in early adolescence, while boys do not develop higher symptom levels until late adolescence [107]. Lastly, the finding that girls reported more trait and state rumination is consistent with previous findings that girls demonstrate greater ruminative response style tendencies beginning in early adolescence [30, 108]. These findings underline the necessity to take into account gender and age differences when studying ER strategies and depressive symptoms in youth.

To end, it should be acknowledged that in the daily diary study most of the variation in state ER strategies occurred between youth, rather than within youth. This is in contrast with previous studies which found most of the variation in state ER strategies to occur across different situations or days (within individuals), rather than across individuals (between individuals) in (young) adult samples when using the ESM [57] or a daily diary approach [109]. This led researchers to conclude that state ER strategy selection may be driven more by contextual factors such as the intensity of the emotion eliciting event [110] than by enduring (temperamental) characteristics [57, 109] and that individuals may adapt their state ER strategy use depending on situational demands [61, 111]. However, our finding that most of the variation in state ER strategies occurred between youth, rather than within youth, is consistent with the trait approach to ER and lines up with the findings of our cross-sectional study as well as with previous research suggesting that there are also stable differences between individuals in the ER strategies they tend to use [55]. While these findings may seem contradictory at first, they are not necessarily mutually exclusive. As stated by [57] both the situation and the person may play a role in the use of ER strategies. More specifically, it could be that reactive temperament traits contribute to defining one’s habitual ER repertoire (trait ER strategies), whereas in daily life also other factors, such as contextual factors, stressors, emotional intensity, and/or personal goals may be decisive in determining which strategy of the ER repertoire is used in a given situation (state ER strategies). In addition, it may be that differences in the design of previous studies may have contributed to explaining the differences in the variance partitioning. As an illustration, in the study of [57], participants were prompted ten times a day to report their use of several ER strategies since the previous sampling moment. Such an ESM design provides different assessments during one day, whereas end-of-day studies only provide one (retrospective) assessment. This may explain why more within-variation was found in the [57] studies. Similarly, the age difference between the sample of our study and the samples included in the study of [57] (participants aged 18 to 35) and [109] (first year college students; *M_age_* = 18 years) may also provide an explanation for the discrepancy in the findings. Since previous research has shown that adolescents tend to use less cognitive ER strategies than adults [47], it may be that the (young) adults included in previous studies have more advanced ER strategies at their disposal in comparison to the children and adolescents included in the current study. Likewise, youth gradually develop the cognitive maturity needed to adopt ER strategies that require more cognitive resources *throughout* the adolescent phase [59, 60] Consequently, more complex cognitive ER strategies such as positive refocusing may be less accessible in the younger part of our sample as cognitive control functions may not be fully developed yet [104, 112]. However, since we controlled for age in all analyses, it is less likely that age-differences in ER strategies may have confounded the results in the current study. Lastly, (young) adults may shift more between ER strategies depending on the situational demands, whereas in youth, the ER repertoire may still be more limited and ER choice may be more dependent on one’s reactive temperament. Future research will need to clarify which of the explanations listed above holds true.

### Clinical implications

First, the finding that NE and PE each provide unique pathways in predicting concurrent depressive symptomatology in average youth suggests that (preventive) ER interventions may need to be personalized in order to adequately target the relevant pathway for each individual. More specifically, the cross-sectional findings suggest that some individuals will benefit from focusing on the reduction of maladaptive ER strategies such as trait rumination [113] while others will profit from learning new adaptive ER strategies such as trait positive refocusing [114]. Although our findings are limited to a community sample of youth, this call for personalized care is also emphasized in clinical studies providing strong indications that different pathways of emotional dynamics may underlie depressive symptomatology [94]. Additional exploratory analyses in the subgroup of youth reporting severe symptomatology, to explore the generalizability of our findings, suggest that clinical youth will especially profit from focusing on the reduction of trait rumination. A finding that is consistent with the theoretical propositions following from integrated models of depression.

Second, our finding that PE–but not NE–significantly predicted daily depressive symptoms in average youth suggests that it may be important for prevention programs to not only focus on alleviating negative emotions but also on enhancing positive emotions by making youth low in PE aware that positive emotions and their consequent upward spirals can be self-generated [62]. Improving positive emotional functioning may not only alleviate symptoms, but also enhance resilience [115].

### Limitations and future directions

Several limitations of the current study warrant discussion. First, the current study exclusively depends on self-report measures. Such an approach is susceptible to common method bias, social desirability bias, and memory bias [116, 117]. Another potential concern of self-report studies involving temperament and depressive symptoms is that symptom levels may have influenced how youth answered items on the self-report measure assessing temperament. For example, youth experiencing depressive symptoms may perceive themselves as experiencing more negative and less positive emotions in general as a result of their current emotional problems, such as sad mood or the inability to experience positive emotions. Researchers have stated that ER may frequently function automatically, outside of conscious awareness [118, 119]. When relying on self-report measures one can only investigate ER strategies that are used consciously, since these strategies are accessible for introspection. Therefore, future research should aim at investigating how automatic ER processes can be reliably measured and how they influence emotional functioning in everyday life. Second, we did not control for (hypo)manic symptoms, neither the presence of other internalizing problems such as symptoms of anxiety. Since there is a high comorbidity amongst these problems [120], further research should also include measures assessing these symptoms in order to clarify whether the found results are specific to depression. Third, results are based on data of a convenience sample of Belgian–Flemish youth and may not be generalizable to Western youth or other (clinical) population groups. While research in a general population sample such as ours serves as a starting point to explore the mechanisms underlying the emotional dynamics in the flow of everyday life, future research in at-risk and clinical population samples is indicated.

Another drawback of the current study is that we only selected two ER strategies: rumination and positive refocusing. Yet, more ER strategies exist. For example, depression has been related to problems with problem solving (i.e., cognitive and behavioural processes by which an individual attempts to identify or discover effective or adaptive solutions for stressful problems [121]) and reappraisal (i.e., reinterpreting the meaning or value of a negative event [7]) as well as the use of maladaptive ER strategies such as avoiding or suppressing negative emotions [38]. On a related note, it has been suggested that youth may often use multiple ER strategies in parallel [32, 122]. An important avenue for future research will thus be to investigate a more exhaustive selection of ER strategies simultaneously, instead of studying ER strategies in isolation, to further disentangle the role of ER strategies for experiencing depressive symptoms in everyday life.

Third, the findings pertaining trait ER strategies are limited by a cross-sectional design that cautions against causal inference. Also, relying on cross-sectional data may yield biased estimates of the longitudinal relation between temperament, trait ER strategies, and depressive symptoms (see [123]). As a result, it cannot be ruled out that other pathways exist. For example, depressive symptoms may also lead youth to ruminate even more, which in turn increases negative affect. Furthermore, difficulties in trait ER strategies may lead to more depressive symptoms because over time, trait rumination increases negative affect. Previous research has shown the relations between ER strategies and psychological adjustment to be mediated by experienced affect [57]. Unfortunately, we did not look at the immediate effects of the state ER strategies on daily affect in the daily diary study. Given the dearth of prior prospective designs, especially in youth, another challenge for future research is to investigate the relations among reactive temperament traits, state ER strategies, symptoms, as well as negative and positive affect longitudinally to disentangle how ER strategies truly relate to the different aspects of emotional functioning.

Fourth, several drawbacks pertain to the use of a 7-day daily diary design in the current study. Even though this methodology is considered to be more ecologically valid than retrospective questionnaires, it remains sensitive to memory biases. Moreover, end-of-day diaries ask youth to what extent they used a certain ER strategy or whether they experienced depressive symptoms over the course of an entire day, which does not offer the possibility to examine how the use of a certain ER strategy is followed by a change in depressive symptoms from one situation to the next. One way to overcome these shortcomings is to use the ESM [124], which may provide a more powerful design to address the dynamic relations between temperament, state ER strategies, and symptoms over a longer period of time. This will be an important future direction for research.

Fifth, we did not include information about the situations in which the state ER strategies were generally applied and the goals youth were trying to achieve. However, in daily life, ER often occurs in social contexts [125] or in response to stress [126], and personal goals are thought to play a defining role in state ER strategies [125]. An important avenue for future research is to study when and how different ER strategies are used by concurrently assessing the context, personal stressors, and personal goals.

Furthermore, to make the design less burdensome, we asked participants to fill out a diary for seven days, which may be a relatively short period to capture meaningful fluctuations in state ER strategies and depressive symptoms, as they may be relatively stable in the short-term. Future research should therefore include longer time frames to enhance assessment of fluctuations in these variables and study the complex dynamics over a longer period of time. The obtained relations in the current daily diary study may change dependent on whether a shorter versus a longer time frame is used.

Lastly, all participants were instructed to start on a Monday to ensure standardization. The down-side of this methodological consideration is however that we cannot rule out the possibility that the psychological effects of the day of the week (see [127]) may have contributed to the current study’s findings. Relatedly, the random slope models in the daily diary analyses showed that depressive symptoms, state rumination, and state positive refocusing all decreased from Monday to Sunday. Whereas this may be a reflection of the stress-levels related to the day of the week [128] we cannot rule out the possibility that measurement reactivity, which is common in daily diary research, may have contributed to our results [129].

Despite these limitations, this study has several notable strengths. First, we used both trait and state assessments of ER strategies and depressive symptoms in a large sample of youth to investigate both the general and day-to-day relations among temperament, ER strategies, and depressive symptoms more thoroughly. Second, unlike the majority of studies linking NE to rumination, we also included PE and an adaptive ER strategy, positive refocusing, in the current study thereby shedding light on the important differences in the dynamics underlying the reactive temperament–depression relation that have been overlooked in studies excluding the PE dimension.

### Conclusion

To conclude, the results of the current study contribute to a more nuanced understanding of the associations between reactive temperament traits, ER strategies, and depressive symptoms in youth by investigating their general as well as their day-to-day associations. The cross-sectional results provide preliminary evidence for integrated models of depression positing that NE and PE each provide distinct and unique pathways in defining one’s vulnerability to depression through influencing one’s trait ER strategies, whereas the daily diary results suggest that in daily life the relations among temperament and state ER strategies may be less clear-cut.

## References

[1] Balazs J. Adolescent Subthreshold Depression and Anxiety. European Psychiatry. 2013;28. WOS:000335460602023.

[2] Burcusa SL, Iacono WG. Risk for recurrence in depression. Clinical Psychology Review. 2007;27(8):959–85. 10.1016/j.cpr.2007.02.005 WOS:000251126200006. 17448579 PMC2169519

[3] Copeland WE, Shanahan L, Costello EJ, Angold A. Childhood and Adolescent Psychiatric Disorders as Predictors of Young Adult Disorders. Archives of General Psychiatry. 2009;66(7):764–72. WOS:000267720200011. 10.1001/archgenpsychiatry.2009.85 19581568 PMC2891142

[4] Nock MK, Green JG, Hwang I, McLaughlin KA, Sampson NA, Zaslavsky AM, et al Prevalence, correlates, and treatment of lifetime suicidal behavior among adolescents: results from the National Comorbidity Survey Replication Adolescent Supplement. JAMA psychiatry. 2013;70(3):300–10. 10.1001/2013.jamapsychiatry.55 23303463 PMC3886236

[5] Hyde JS, Mezulis AH, Abramson LY. The ABCs of depression: integrating affective, biological, and cognitive models to explain the emergence of the gender difference in depression. Psychological review. 2008;115(2):291 10.1037/0033-295X.115.2.291 18426291

[6] Yap MB, Allen NB, Sheeber L. Using an emotion regulation framework to understand the role of temperament and family processes in risk for adolescent depressive disorders. Clinical child and family psychology review. 2007;10(2):180–96. 10.1007/s10567-006-0014-0 17265137

[7] Gross JJ. Handbook of emotion regulation: Guilford publications; 2013.

[8] Rothbart M, Posner M, Hershey K. Developmental psychopathology: Theory and methods. 1995.

[9] Rothbart M, Bates J. Temperament (In Damon W., Lerner R., & Eisenberg N.(Eds.). Handbook of child psychology. Social, emotional, and personality development (Vol. 3, pp. 99–166). New York: Wiley; 2006.

[10] Muris P. Unique and interactive effects of neuroticism and effortful control on psychopathological symptoms in non-clinical adolescents. Personality and Individual Differences. 2006;40(7):1409–19. 10.1016/j.paid.2005.12.001 WOS:000237166700008.

[11] Muris P, Meesters C, Blijlevens P. Self-reported reactive and regulative temperament in early adolescence: Relations to internalizing and externalizing problem behavior and "Big Three" personality factors. Journal of Adolescence. 2007;30(6):1035–49. 10.1016/j.adolescence.2007.03.003 WOS:000251675100009. 17467051

[12] Rothbart MK, Ahadi SA, Evans DE. Temperament and personality: Origins and outcomes. Journal of Personality and Social Psychology. 2000;78(1):122–35. 10.1037//0022-3514.78.1.122 WOS:000084760900010. 10653510

[13] Mezulis AH, Rudolph ME. Pathways linking temperament and depressive symptoms: A short-term prospective diary study among adolescents. Cognition & Emotion. 2012;26(5):950–60. 10.1080/02699931.2012.665027 WOS:000306542100016. 22650304

[14] Verstraeten K, Vasey MW, Raes F, Bijttebier P. Temperament and risk for depressive symptoms in adolescence: Mediation by rumination and moderation by effortful control. Journal of abnormal child psychology. 2009;37(3):349–61. 10.1007/s10802-008-9293-x 19107592

[15] Wetter EK, Hankin BL. Mediational Pathways Through Which Positive and Negative Emotionality Contribute to Anhedonic Symptoms of Depression: A Prospective Study of Adolescents. Journal of Abnormal Child Psychology. 2009;37(4):507–20. 10.1007/s10802-009-9299-z WOS:000264879800005. 19184402 PMC2742579

[16] Lonigan CJ, Phillips BM, Hooe ES. Relations of positive and negative affectivity to anxiety and depression in children: evidence from a latent variable longitudinal study. Journal of consulting and clinical psychology. 2003;71(3):465 10.1037/0022-006x.71.3.465 12795571

[17] Clark LA, Watson D. Tripartite Model of Anxiety and Depression—Psychometric Evidence and Taxonomic Implications. Journal of Abnormal Psychology. 1991;100(3):316–36. 10.1037//0021-843x.100.3.316 WOS:A1991FY37600011. 1918611

[18] Khazanov GK, Ruscio AM. Is low positive emotionality a specific risk factor for depression? A meta-analysis of longitudinal studies. 2016. 10.1037/bul0000059 PMC5110375 27416140

[19] Fredrickson BL. The Role of Positive Emotions in Positive Psychology: The Broaden-and-Build Theory of Positive Emotions. The American psychologist. 2001;56(3):218–26. PMC3122271. 10.1037//0003-066x.56.3.218 11315248 PMC3122271

[20] Van Beveren M-L, McIntosh K, Vandevivere E, Wante L, Vandeweghe L, Van Durme K, et al Associations Between Temperament, Emotion Regulation, and Depression in Youth: The Role of Positive Temperament. Journal of Child and Family Studies. 2016;25(6):1954–68.

[21] Vasey MW, Harbaugh CN, Lonigan CJ, Phillips BM, Hankin BL, Willem L, et al Dimensions of temperament and depressive symptoms: Replicating a three-way interaction. Journal of Research in Personality. 2013;47(6):908–21. 10.1016/j.jrp.2013.09.001 24493906 PMC3908677

[22] Joiner TE, Lonigan CJ. Tripartite model of depression and anxiety in youth psychiatric inpatients: Relations with diagnostic status and future symptoms. Journal of Clinical Child Psychology. 2000;29(3):372–82. 10.1207/S15374424JCCP2903_8 10969421

[23] Thompson RA. The development of the person: Social understanding, relationships, conscience, self. Handbook of child psychology. 2006.

[24] Yap MB, Allen NB, O'Shea M, Di Parsia P, Simmons JG, Sheeber L. Early adolescents' temperament, emotion regulation during mother–child interactions, and depressive symptomatology. Development and psychopathology. 2011;23(1):267–82. 10.1017/S0954579410000787 21262053

[25] Mezulis A, Simonson J, McCauley E, Vander Stoep A. The association between temperament and depressive symptoms in adolescence: Brooding and reflection as potential mediators. Cognition & emotion. 2011;25(8):1460–70. 21432637 10.1080/02699931.2010.543642 PMC3626415

[26] Hudson MR, Harding KA, Mezulis A. Dampening and brooding jointly link temperament with depressive symptoms: A prospective study. Personality and Individual Differences. 2015;83:249–54. 10.1016/j.paid.2015.04.025 WOS:000356642400044.

[27] Harding KA, Hudson MR, Mezulis A. Cognitive mechanisms linking low trait positive affect to depressive symptoms: A prospective diary study. Cognition and emotion. 2014;28(8):1502–11. 10.1080/02699931.2014.889661 24552238

[28] Van Beveren ML, Harding K, Beyers W, Braet C. Don't worry, be happy: The role of positive emotionality and adaptive emotion regulation strategies for youth depressive symptoms. British Journal of Clinical Psychology. 2017. 10.1111/bjc.12151 28833279

[29] Nolen-Hoeksema S. Responses to depression and their effects on the duration of depressive episodes. Journal of abnormal psychology. 1991;100(4):569 10.1037//0021-843x.100.4.569 1757671

[30] Nolen-Hoeksema S, Girgus JS. The emergence of gender differences in depression during adolescence. Psychological bulletin. 1994;115(3):424 10.1037/0033-2909.115.3.424 8016286

[31] Gross JJ. Emotion regulation: Past, present, future. Cognition & Emotion. 1999;13(5):551–73. 10.1080/026999399379186 WOS:000082383300006.

[32] Gross JJ, Thompson RA. Emotion regulation: Conceptual foundations. 2007.

[33] Lane RD, Schwartz GE. Levels of emotional awareness: A cognitive-developmental theory and its application to psychopathology. The American journal of psychiatry. 1987. 10.1176/ajp.144.2.133 3812780

[34] Berking M, Whitley B. Affect regulation training. Handbook of emotion regulation. 2014;2.

[35] Nigg JT. Annual Research Review: On the relations among self‐regulation, self‐control, executive functioning, effortful control, cognitive control, impulsivity, risk‐taking, and inhibition for developmental psychopathology. Journal of child psychology and psychiatry. 2017;58(4):361–83. 10.1111/jcpp.12675 28035675 PMC5367959

[36] Koole SL. The psychology of emotion regulation: An integrative review Cognition and emotion: Psychology press; 2010 p. 138–77.

[37] Schäfer JÖ, Naumann E, Holmes EA, Tuschen-Caffier B, Samson AC. Emotion regulation strategies in depressive and anxiety symptoms in youth: a meta-analytic review. Journal of youth and adolescence. 2017;46(2):261–76. 10.1007/s10964-016-0585-0 27734198

[38] Aldao A, Nolen-Hoeksema S, Schweizer S. Emotion-regulation strategies across psychopathology: A meta-analytic review. Clinical Psychology Review. 2010;30(2):217–37. 10.1016/j.cpr.2009.11.004 WOS:000274319600007. 20015584

[39] Aldao A, Nolen-Hoeksema S. Specificity of cognitive emotion regulation strategies: A transdiagnostic examination. Behaviour Research and Therapy. 2010;48(10):974–83. 10.1016/j.brat.2010.06.002 WOS:000282406300005. 20591413

[40] Hyde JS, Mezulis AH, Abramson LY. The ABCs of depression: Integrating affective, biological, and cognitive models to explain the emergence of the gender difference in depression. Psychological Review. 2008;115(2):291–313. 10.1037/0033-295X.115.2.291 WOS:000255118800001. 18426291

[41] Mezulis AH, Priess HA, Hyde JS. Rumination mediates the relationship between infant temperament and adolescent depressive symptoms. Depression research and treatment. 2010;2011. 10.1155/2011/487873 PMC2989647 21151502

[42] Peterson C, Seligman ME. Causal explanations as a risk factor for depression: Theory and evidence. Psychological review. 1984;91(3):347 6473583

[43] Fredrickson BL, Branigan C. Positive emotions broaden the scope of attention and thought‐action repertoires. Cognition & emotion. 2005;19(3):313–32. 21852891 10.1080/02699930441000238 PMC3156609

[44] Fredrickson BL, Joiner T. Positive emotions trigger upward spirals toward emotional well-being. Psychological science. 2002;13(2):172–5. 10.1111/1467-9280.00431 11934003

[45] Fredrickson BL. The role of positive emotions in positive psychology: The broaden-and-build theory of positive emotions. American psychologist. 2001;56(3):218 10.1037//0003-066x.56.3.218 11315248 PMC3122271

[46] Folkman S, Moskowitz JT. Positive affect and the other side of coping. American psychologist. 2000;55(6):647 10.1037//0003-066x.55.6.647 10892207

[47] Garnefski N, Kraaij V. Relationships between cognitive emotion regulation strategies and depressive symptoms: A comparative study of five specific samples. Personality and Individual Differences. 2006;40(8):1659–69. 10.1016/j.paid.2005.12.009 WOS:000237901000014.

[48] Quoidbach J, Berry EV, Hansenne M, Mikolajczak M. Positive emotion regulation and well-being: Comparing the impact of eight savoring and dampening strategies. Personality and individual differences. 2010;49(5):368–73.

[49] Raes F, Daems K, Feldman GC, Johnson SL, Van Gucht D. A psychometric evaluation of the Dutch version of the responses to positive affect questionnaire. Psychologica Belgica. 2009;49(4):293.

[50] Werner-Seidler A, Banks R, Dunn BD, Moulds ML. An investigation of the relationship between positive affect regulation and depression. Behaviour research and therapy. 2013;51(1):46–56. 10.1016/j.brat.2012.11.001 23178678

[51] Garnefski N, Kraaij V, Spinhoven P. Negative life events, cognitive emotion regulation and emotional problems. Personality and Individual Differences. 2001;30(8):1311–27. 10.1016/S0191-8869(00)00113-6 WOS:000168777000004.

[52] Braet C, Theuwis L, Van Durme K, Vandewalle J, Vandevivere E, Wante L, et al Emotion Regulation in Children with Emotional Problems. Cognitive Ther Res. 2014;38(5):493–504. 10.1007/s10608-014-9616-x WOS:000342131800002.

[53] Mascha C, Bockting CL, Koeter MW, Schene AH. Prediction of recurrence in recurrent depression: a 5.5-year prospective study. The Journal of clinical psychiatry. 2010;71(8):984–91. 10.4088/JCP.08m04858blu 20797379

[54] Verstraeten K, Bijttebier P, Vasey MW, Raes F. Specificity of worry and rumination in the development of anxiety and depressive symptoms in children. British Journal of Clinical Psychology. 2011;50(4):364–78. 10.1348/014466510X532715 22003947

[55] Gross JJ, John OP. Individual differences in two emotion regulation processes: Implications for affect, relationships, and well-being. Journal of Personality and Social Psychology. 2003;85(2):348–62. 10.1037/0022-3514.85.2.348 WOS:000184523900012. 12916575

[56] Tugade MM, Fredrickson BL. Regulation of positive emotions: Emotion regulation strategies that promote resilience. Journal of Happiness Studies. 2007;8(3):311–33.

[57] Brans K, Koval P, Verduyn P, Lim YL, Kuppens P. The regulation of negative and positive affect in daily life. Emotion. 2013;13(5):926 10.1037/a0032400 23731436

[58] Calkins SD, Mackler JS. Temperament, emotion regulation, and social development. Social development: Relationships in infancy, childhood, and adolescence. 2011:44–70.

[59] Eisenberg N, Morris AS. Children's emotion-related regulation. 2002. 12402675

[60] Ahmed SP, Bittencourt-Hewitt A, Sebastian CL. Neurocognitive bases of emotion regulation development in adolescence. Developmental Cognitive Neuroscience. 2015;15:11–25. 10.1016/j.dcn.2015.07.006 WOS:000364537300002. 26340451 PMC6989808

[61] Sheppes G, Scheibe S, Suri G, Gross JJ. Emotion-Regulation Choice. Psychological Science. 2011;22(11):1391–6. 10.1177/0956797611418350 WOS:000300826400006. 21960251

[62] Garland EL, Fredrickson B, Kring AM, Johnson DP, Meyer PS, Penn DL. Upward spirals of positive emotions counter downward spirals of negativity: Insights from the broaden-and-build theory and affective neuroscience on the treatment of emotion dysfunctions and deficits in psychopathology. Clinical psychology review. 2010;30(7):849–64. 10.1016/j.cpr.2010.03.002 20363063 PMC2908186

[63] Vasey MW, Harbaugh CN, Fisher LB, Heath JH, Hayes AF, Bijttebier P. Temperament synergies in risk for and protection against depressive symptoms: A prospective replication of a three-way interaction. Journal of Research in Personality. 2014;53:134–47.

[64] Van Beveren M-L, Mezulis A, Wante L, Braet C. Joint contributions of negative emotionality, positive emotionality, and effortful control on depressive symptoms in youth. Journal of Clinical Child & Adolescent Psychology. 2016:1–12. 10.1080/15374416.2016.1233499 27805840

[65] Thayer JF, Rossy LA, Ruiz-Padial E, Johnsen BH. Gender differences in the relationship between emotional regulation and depressive symptoms. Cognitive Therapy and Research. 2003;27(3):349–64.

[66] Hollingshead AB. Four factor index of social status. 1975.

[67] Timbremont B, Braet C, Dreessen L. Assessing depression in youth: relation between the Children's Depression Inventory and a structured interview. Journal of Clinical Child and Adolescent Psychology. 2004;33(1):149–57. 10.1207/S15374424JCCP3301_14 15028549

[68] Mabbe E, Soenens B, Vansteenkiste M, van der Kaap-Deeder J, Mouratidis A. Day-to-day variation in autonomy-supportive and psychologically controlling parenting: The role of parents’ daily experiences of need satisfaction and need frustration. Parenting. 2018;18(2):86–109.

[69] Vandewalle J, Mabbe E, Debeuf T, Braet C, Moens E. The Daily Relation between Parental Rejection and Emotional Eating in Youngsters: A Diary Study. Frontiers in psychology. 2017;8:691 10.3389/fpsyg.2017.00691 28553239 PMC5425587

[70] Hruska LC, Zelic KJ, Dickson KS, Ciesla JA. Adolescents' co‐rumination and stress predict affective changes in a daily‐diary paradigm. International Journal of Psychology. 2017;52(5):372–80. 10.1002/ijop.12227 26493516

[71] Little RJ. A test of missing completely at random for multivariate data with missing values. Journal of the American Statistical Association. 1988;83(404):1198–202.

[72] Bollen KA. Structural equations with latent variables: John Wiley & Sons; 2014.

[73] Schafer JL. Analysis of incomplete multivariate data: CRC press; 1997.

[74] Laurent J, Catanzaro SJ, Joiner TE Jr, Rudolph KD, Potter KI, Lambert S, et al A measure of positive and negative affect for children: scale development and preliminary validation. Psychological assessment. 1999;11(3):326.

[75] Verstraeten K, Vasey MW, Raes F, Bijttebier P. The mediational role of responses to positive affect in the association between temperament and (hypo) manic symptoms in children. Cognitive therapy and research. 2012;36(6):768–75.

[76] Van Beveren M-L, Mueller SC, Braet C. Emotion dysregulation, temperamental vulnerability, and parental depression in adolescents: Correspondence between physiological and informant-report measures. Development and psychopathology. 2019:1–13. 10.1017/S0954579419000567 31046860

[77] Cannon MF, Weems CF. Do anxiety and depression cluster into distinct groups?: A test of tripartite model predictions in a community sample of youth. Depression and Anxiety. 2006;23(8):453–60. 10.1002/da.20215 16845650

[78] Grob A, Smolenski C. Fragebogen zur Erhebung der Emotionsregulation bei Kindern und Jugendlichen (FEEL-KJ): Verlag Hans Huber; 2005.

[79] Braet C, Cracco E, Theuwis L, Grob A, Smolenski C. FEEL-KJ: vragenlijst over emotieregulatie bij kinderen en jongeren: Hogrefe Amsterdam; 2013.

[80] Schmitt K, Gold A, Rauch W. [Deficient adaptive regulation of emotion in children with ADHD]. Zeitschrift fur Kinder-und Jugendpsychiatrie und Psychotherapie. 2012;40(2):95–102; quiz -3. 10.1024/1422-4917/a000156 22354493

[81] Lewis CA, Loewenthal K. An introduction to psychological tests and scales: Psychology Press; 2015.

[82] Kovacs M. Children's Depression Inventory (CDI) manual. New York: Multi-Health Systems; 1992.

[83] Timbremont B, Braet C. Children's Depression Inventory, Nederlandstalige versie (Children's Depression Inventory, Dutch version). Lisse: Swets & Zeitlinger; 2002.

[84] Martens MP, Parker JC, Smarr KL, Hewett JE, Ge B, Slaughter JR, et al Development of a shortened Center for Epidemiological Studies Depression scale for assessment of depression in Rheumatoid Arthritis. Rehabilitation Psychology. 2006;51(2):135.

[85] Radloff LS. The CES-D scale: A self-report depression scale for research in the general population. Applied psychological measurement. 1977;1(3):385–401.

[86] Faulstich ME, Carey MP, Ruggiero L, Enyart P, Gresham F. Assessment of depression in childhood and adolescence: an evaluation of the Center for Epidemiological Studies Depression Scale for Children (CES-DC). American Journal of Psychiatry. 1986;143(8):1024–7. 10.1176/ajp.143.8.1024 . 3728717

[87] Preacher KJ, Hayes AF. Asymptotic and resampling strategies for assessing and comparing indirect effects in multiple mediator models. Behavior Research Methods. 2008;40(3):879–91. 10.3758/Brm.40.3.879 ISI:000257991700027. 18697684

[88] Hayes AF. Introduction to mediation, moderation, and conditional process analysis: A regression-based approach: Guilford Press; 2013.

[89] Rasbash J, Charlton C, Browne WJ, Healy M, Cameron B. MLwiN Version 2.1 Centre for multilevel modelling, University of Bristol 2009:1.

[90] Nezlek JB. Multilevel modeling for social and personality psychology: SAGE Publications Ltd; 2011.

[91] Enders CK, Tofighi D. Centering predictor variables in cross-sectional multilevel models: a new look at an old issue. Psychological methods. 2007;12(2):121 10.1037/1082-989X.12.2.121 17563168

[92] Laurent J, Joiner TE Jr, Catanzaro SJ. Positive affect, negative affect, and physiological hyperarousal among referred and nonreferred youths. Psychological Assessment. 2011;23(4):945 10.1037/a0024080 21744972

[93] Kercher AJ, Rapee RM, Schniering CA. Neuroticism, life events and negative thoughts in the development of depression in adolescent girls. Journal of Abnormal Child Psychology. 2009;37(7):903–15. 10.1007/s10802-009-9325-1 19437113

[94] Bos F, Blaauw F, Snippe E, Van der Krieke L, De Jonge P, Wichers M. Exploring the emotional dynamics of subclinically depressed individuals with and without anhedonia: An experience sampling study. Journal of affective disorders. 2018;228:186–93. 10.1016/j.jad.2017.12.017 29253685

[95] Fredrickson BL, Cohn MA, Coffey KA, Pek J, Finkel SM. Open hearts build lives: positive emotions, induced through loving-kindness meditation, build consequential personal resources. Journal of personality and social psychology. 2008;95(5):1045 10.1037/a0013262 18954193 PMC3156028

[96] Peeters F, Berkhof J, Rottenberg J, Nicolson NA. Ambulatory emotional reactivity to negative daily life events predicts remission from major depressive disorder. Behaviour research and therapy. 2010;48(8):754–60. 10.1016/j.brat.2010.04.008 20537317

[97] Rottenberg J, Gross JJ, Gotlib IH. Emotion context insensitivity in major depressive disorder. Journal of abnormal psychology. 2005;114(4):627 10.1037/0021-843X.114.4.627 16351385

[98] Forbes EE, Dahl RE. Research review: altered reward function in adolescent depression: what, when and how? Journal of Child Psychology and Psychiatry. 2012;53(1):3–15. 10.1111/j.1469-7610.2011.02477.x 22117893 PMC3232324

[99] Eshel N, Roiser JP. Reward and punishment processing in depression. Biological psychiatry. 2010;68(2):118–24. 10.1016/j.biopsych.2010.01.027 20303067

[100] Henriques JB, Davidson RJ. Decreased responsiveness to reward in depression. Cognition & Emotion. 2000;14(5):711–24.

[101] Tan PZ, Forbes EE, Dahl RE, Ryan ND, Siegle GJ, Ladouceur CD, et al Emotional reactivity and regulation in anxious and nonanxious youth: A cell‐phone ecological momentary assessment study. Journal of Child Psychology and Psychiatry. 2012;53(2):197–206. 10.1111/j.1469-7610.2011.02469.x 22176136 PMC3258378

[102] Balázs J, Miklósi M, Keresztény Á, Hoven CW, Carli V, Wasserman C, et al Adolescent subthreshold‐depression and anxiety: Psychopathology, functional impairment and increased suicide risk. Journal of child psychology and psychiatry. 2013;54(6):670–7. 10.1111/jcpp.12016 23330982

[103] Jose PE, Brown I. When does the gender difference in rumination begin? Gender and age differences in the use of rumination by adolescents. Journal of Youth and Adolescence. 2008;37(2):180–92.

[104] Steinberg L. Cognitive and affective development in adolescence. Trends in cognitive sciences. 2005;9(2):69–74. 10.1016/j.tics.2004.12.005 15668099

[105] Hankin BL, Abramson LY, Moffitt TE, Silva PA, McGee R, Angell KE. Development of depression from preadolescence to young adulthood: emerging gender differences in a 10-year longitudinal study. Journal of abnormal psychology. 1998;107(1):128 10.1037//0021-843x.107.1.128 9505045

[106] Wichstrøm L. The emergence of gender difference in depressed mood during adolescence: the role of intensified gender socialization. Developmental psychology. 1999;35(1):232 9923478

[107] Salk RH, Petersen JL, Abramson LY, Hyde JS. The contemporary face of gender differences and similarities in depression throughout adolescence: Development and chronicity. Journal of affective disorders. 2016;205:28–35. 10.1016/j.jad.2016.03.071 27391269 PMC5468750

[108] Hankin BL, Abramson LY. Measuring cognitive vulnerability to depression in adolescence: Reliability, validity, and gender differences. Journal of clinical child and adolescent psychology. 2002;31(4):491–504. 10.1207/S15374424JCCP3104_8 12402568

[109] English T, Lee IA, John OP, Gross JJ. Emotion regulation strategy selection in daily life: The role of social context and goals. Motivation and Emotion. 2016:1–13. 10.1007/s11031-016-9597-z PMC5482525 28652647

[110] Luminet O, Zech E, Rimé B, Wagner H. Predicting cognitive and social consequences of emotional episodes: The contribution of emotional intensity, the five factor model, and alexithymia. Journal of Research in Personality. 2000;34(4):471–97.

[111] Cheng C. Assessing coping flexibility in real-life and laboratory settings: a multimethod approach. Journal of personality and social psychology. 2001;80(5):814 10.1037//0022-3514.80.5.814 11374752

[112] Ahmed S, Bittencourt-Hewitt A, Sebastian C. Neurocognitive bases of emotion regulation development in adolescence. Dev. Cogn. Neurosci 15, 11–25. 2015 10.1016/j.dcn.2015.07.006 26340451 PMC6989808

[113] Watkins E, Scott J, Wingrove J, Rimes K, Bathurst N, Steiner H, et al Rumination-focused cognitive behaviour therapy for residual depression: A case series. Behaviour research and therapy. 2007;45(9):2144–54. 10.1016/j.brat.2006.09.018 17367751

[114] Hannesdottir DK, Ollendick TH. The role of emotion regulation in the treatment of child anxiety disorders. Clinical Child and Family Psychology Review. 2007;10(3):275–93. 10.1007/s10567-007-0024-6 17705098

[115] Ehrenreich JT, Fairholme CP, Buzzella BA, Ellard KK, Barlow DH. The role of emotion in psychological therapy. Clinical Psychology: Science and Practice. 2007;14(4):422–8. 10.1111/j.1468-2850.2007.00102.x PMC2562704 18843381

[116] Schwarz N. Self-reports: How the questions shape the answers. American psychologist. 1999;54(2):93.

[117] Scollon CN, Prieto C-K, Diener E. Experience sampling: promises and pitfalls, strength and weaknesses Assessing well-being: Springer; 2009 p. 157–80.

[118] Gyurak A, Gross JJ, Etkin A. Explicit and implicit emotion regulation: a dual-process framework. Cognition and emotion. 2011;25(3):400–12. 10.1080/02699931.2010.544160 21432682 PMC3280343

[119] Mauss IB, Bunge SA, Gross JJ. Automatic emotion regulation. Social and Personality Psychology Compass. 2007;1(1):146–67.

[120] Axelson DA, Birmaher B. Relation between anxiety and depressive disorders in childhood and adolescence. Depression and anxiety. 2001;14(2):67–78. 11668659 10.1002/da.1048

[121] D'Zurilla TJ, Nezu AM, Maydeu-Olivares A. Social problem solving: Theory and assessment. 2004.

[122] Webb TL, Miles E, Sheeran P. Dealing with feeling: a meta-analysis of the effectiveness of strategies derived from the process model of emotion regulation. Psychological bulletin. 2012;138(4):775 10.1037/a0027600 22582737

[123] Maxwell SE, Cole DA, Mitchell MA. Bias in cross-sectional analyses of longitudinal mediation: Partial and complete mediation under an autoregressive model. Multivariate Behavioral Research. 2011;46(5):816–41. 10.1080/00273171.2011.606716 26736047

[124] Bylsma LM, Rottenberg J. Uncovering the dynamics of emotion regulation and dysfunction in daily life with ecological momentary assessment Emotion regulation and well-being: Springer; 2011 p. 225–44.

[125] Gross JJ, Richards JM, John OP. Emotion regulation in everyday life. Emotion regulation in couples and families: Pathways to dysfunction and health. 2006;2006:13–35.

[126] Gross JJ. Antecedent- and response-focused emotion regulation: Divergent consequences for experience, expression, and physiology. Journal of Personality and Social Psychology. 1998;74(1):224–37. 10.1037//0022-3514.74.1.224 WOS:000071543400017. 9457784

[127] Areni CS, Burger M, Zlatevska N. Factors affecting the extent of Monday blues: Evidence from a meta-analysis. Psychological reports. 2011;109(3):723–33. 10.2466/13.20.PR0.109.6.723-733 22420107

[128] van Roekel E, Ha T, Verhagen M, Kuntsche E, Scholte RH, Engels RC. Social stress in early adolescents' daily lives: Associations with affect and loneliness. Journal of adolescence. 2015;45:274–83. 10.1016/j.adolescence.2015.10.012 26545263

[129] Csikszentmihalyi M. Handbook of research methods for studying daily life: Guilford Press; 2011.

